# Plasma Malondialdehyde Reference Interval and Its Associations with Lifestyle Risk Factors in Thai Undergraduate Students: A Single-Center Cross-Sectional Study

**DOI:** 10.3390/diagnostics16172697

**Published:** 2026-08-24

**Authors:** Supranee Kongkham, Kanyawee Chainam, Tikamporn Chobkarn, Thitinan Choechu, Chuthamat Phromthon, Pimchanok Maomeuang, Wiphawan Wasenang

**Affiliations:** 1 Faculty of Allied Health Sciences, Nakhon Ratchasima College, Nakhon Ratchasima 30000, Thailand; 2 Faculty of Public Health, Mahasarakham University, Maha Sarakham 44150, Thailand

**Keywords:** malondialdehyde, oxidative stress, reference interval, young adults, non-communicable diseases, low-grade inflammation, screening biomarker

## Abstract

**Background/Objectives**: Oxidative stress and low-grade chronic inflammation are implicated in the early pathogenesis of non-communicable diseases (NCDs). Malondialdehyde (MDA), a biomarker of lipid peroxidation, may reflect subclinical oxidative stress in apparently healthy young adults. This study aimed to establish a population-specific reference interval for plasma MDA in healthy Thai undergraduate students and evaluate associations with lifestyle risk factors. **Methods**: This cross-sectional study enrolled 141 Thai college students aged 18–27 years. Plasma MDA was measured using the thiobarbituric acid reactive substances (TBARS) assay. A reference interval was determined non-parametrically (P2.5–P97.5) following IFCC recommendations in a reference subgroup (*n* = 94). The 75th percentile (P75) was used as an exploratory cutoff for elevated MDA. Associations between MDA and lifestyle risk factors were assessed using logistic regression and multiple linear regression. **Results**: The plasma MDA reference interval was 1.16–9.23 µmol/L, with P75 = 6.07 µmol/L. Using this cutoff, 25.5% of participants had elevated MDA. High sugar intake was associated with elevated MDA in the P75-based model (adjusted OR = 3.42; 95% CI: 1.08–10.87; *p* = 0.036). In the linear regression model, BMI emerged as independently associated with MDA (*p* = 0.004). **Conclusions**: This study established the first population-specific plasma MDA reference interval for Thai young adults. High sugar intake and BMI emerged as complementary exploratory associations with elevated lipid peroxidation across different models. These findings support plasma MDA as a practical, cost-effective candidate biomarker, providing a basis for further early oxidative stress surveillance and NCD prevention in young populations.

## 1. Introduction

Non-communicable diseases (NCDs), particularly metabolic diseases and atherosclerotic cardiovascular disease (ASCVD), remain the foremost global public health challenge of the twenty-first century. In 2021, NCDs killed at least 43 million people, equivalent to 75% of all non-pandemic-related deaths, with approximately 18 million occurring among individuals younger than 70 years, 82% of whom lived in low- and middle-income countries [1]. While global age-standardized mortality rates have declined overall, the Global Burden of Disease (GBD) 2023 study highlighted that premature NCD mortality and its burden on adolescents and young adults remain significant concerns, particularly in low- and middle-income countries [2]. NCDs are a major contributor to mortality and the overall disease burden in Thailand, accounting for approximately 76% of all deaths, of which 86% occur among individuals aged under 70 years [3]. The burden is escalating: the 7th Thai National Health Examination Survey (NHES VII, 2024–2025) revealed that metabolic syndrome now affects 28% of Thais aged 15 years and above [4], representing a substantial increase from the 23.2% prevalence reported in the 4th NHES in 2009 [5]. Among university students, the prevalence of modifiable NCD risk factors is notably high. A multi-country study across 24 nations found that 15.9% of university students exhibited three or more behavioral NCD risk factors [6], and a cross-sectional study of 485 students found that more than half (51.2%) had four or more concurrent risk factors, with physical inactivity being the most prevalent (89.2%) [7]. Thai undergraduate students exhibit analogous patterns, including unhealthy dietary habits, sedentary lifestyles, sleep deprivation, and psychological stress. This constellation of behaviors collectively promotes oxidative stress, a key upstream mechanism in metabolic dysregulation and ASCVD development.

Oxidative stress arises from an imbalance between reactive oxygen species (ROS) production and antioxidant defense mechanisms [8]. Lifestyle-related triggers, including diets rich in ultra-processed foods, saturated fats, and refined sugars; physical inactivity; sleep disorders; and excessive alcohol intake, promote oxidative stress, mitochondrial dysfunction, and the release of inflammation-inducing signals, sustaining a state of low-grade chronic inflammation [9]. Low-grade chronic inflammation is a persistent, subclinical state arising from failure of the normal inflammatory resolution process and is strongly associated with obesity, metabolic syndrome, type 2 diabetes mellitus, ASCVD, and chronic kidney disease [10]. Importantly, subclinical oxidative stress and early vascular changes, including impaired flow-mediated dilation, have been documented in young adults with unhealthy lifestyle profiles even in the absence of overt metabolic disease [11]. Lipid peroxidation is a principal consequence of oxidative stress, generating reactive aldehydes, particularly malondialdehyde (MDA), a stable, sensitive biomarker of lipid peroxidation and oxidative stress [12]. Elevated MDA has been reported across cardiometabolic conditions including obesity, metabolic syndrome, diabetes mellitus, hypertension, and cardiovascular disease [13]. Beyond its role as a passive marker, MDA actively mediates vascular injury by generating atherogenic MDA-modified LDL, activating pro-inflammatory signaling pathways, and upregulating inflammatory cytokines in lymphocytes, thereby establishing a feedforward loop that sustains low-grade inflammation [14,15].

Assessment of oxidative stress and low-grade inflammation in clinical and epidemiological settings commonly relies on high-sensitivity C-reactive protein (hsCRP), a widely used marker of systemic inflammation. Although hsCRP correlates with oxidative stress, it reflects downstream inflammatory activation rather than upstream oxidative damage itself, and its levels in apparently healthy young individuals often remain within the normal range. This limits its sensitivity for detecting early subclinical lipid peroxidation in this population [16]. Moreover, hsCRP measurement typically requires immunoturbidimetric or high-sensitivity ELISA-based platforms, which may be less accessible in resource-limited settings [17]. Unlike hsCRP, MDA directly measures upstream oxidative damage rather than downstream inflammatory activation, providing mechanistically complementary information on cardiometabolic risk [13], while the TBARS-based assay requires only a spectrophotometer and basic reagents, making it simple, cost-effective, and practical for large-scale screening [17]. Despite its clinical utility, a critical limitation constraining routine MDA application is the absence of validated, population-specific reference intervals. Existing reference values vary substantially by analytical method and population characteristics [18]. The most widely cited plasma MDA reference interval, established in a Danish cohort using HPLC, yielded 0.36–1.24 µmol/L [19], considerably lower than values typically obtained using the TBARS-based spectrophotometric method commonly used in clinical and epidemiological research [20]. As reference intervals are not transferable across methods or populations, values from Western or mixed-age cohorts may not apply to young Asian populations with distinct dietary, body composition, and oxidative stress profiles [18]. To date, no population-specific reference interval for plasma MDA using the TBARS-spectrophotometric method has been established in Thai young adults.

Modern lifestyle patterns among Thai undergraduate students further heighten these concerns. Studies have demonstrated that Thai university students exhibit a high prevalence of modifiable cardiometabolic risk behaviors. These include frequent consumption of ultra-processed foods and sugar-sweetened beverages, physical inactivity, and prolonged sedentary behavior [3,21]. Despite this, existing Thai studies have focused predominantly on conventional parameters such as body mass index (BMI), waist circumference, fasting blood glucose, lipid profiles, and blood pressure. These parameters do not capture upstream oxidative and inflammatory mechanisms that precede clinically detectable metabolic disease [22]. Direct evidence of oxidative stress burden and its relationship with low-grade chronic inflammation in this population therefore remains scarce. Early identification of elevated plasma MDA in relation to specific lifestyle risk factors could provide a timely foundation for risk-targeted health promotion interventions aimed at reducing the future burden of ASCVD among Thai young adults [16].

Therefore, this study aimed to (1) establish a population-specific reference interval for plasma MDA in healthy Thai undergraduate students following International Federation of Clinical Chemistry and Laboratory Medicine (IFCC) recommendations; (2) evaluate plasma MDA levels and their associations with lifestyle risk factors, including high-fat diet, high sodium intake, excessive sugar intake, physical inactivity, inadequate sleep duration, smoking, and alcohol consumption. We hypothesized that unhealthy lifestyle behaviors, particularly high-fat and high-sugar dietary patterns, would be independently associated with elevated plasma MDA, reflecting increased lipid peroxidation and low-grade chronic inflammation linked to early metabolic alterations and future ASCVD risk. The findings are expected to contribute to evidence-based preventive strategies and university-level health promotion programs for reducing long-term NCD risk in Thai young adults.

## 2. Materials and Methods

### 2.1. Study Population and Design

This cross-sectional study enrolled 141 Thai college students aged 18–27 years (20.8 ± 1.7 years). The sample size was calculated a priori based on the prevalence of elevated plasma MDA (10.2%) reported in a Thai population [23], yielding a minimum required sample size of 141 participants. Participants were recruited via voluntary sampling following public announcement of a health screening event between August and September 2025 at Nakhon Ratchasima College, Thailand. Informed consent was obtained from all individuals involved in this study. The study protocol was approved by the Nakhon Ratchasima College Ethics Committee (NMCEC-0057/2567). A healthy reference subgroup (*n* = 94) was selected from the total cohort based on the following inclusion criteria: non-obese (BMI < 25 kg/m^2^) [24], normal waist circumference (women < 80 cm, men < 90 cm) [25], and normal blood pressure (systolic < 140 mmHg and diastolic < 90 mmHg) [26]. Individuals with a history of chronic disease or current use of medications known to influence oxidative stress levels were excluded from this subgroup. Of the 141 enrolled participants, 94 met all criteria and were therefore selected to establish the plasma malondialdehyde (MDA) reference interval.

### 2.2. Data Collection

Body weight and height were measured with participants barefoot and in light clothing. Waist circumference was measured at the midpoint between the lowest rib and iliac crest. Blood pressure was measured in triplicate at 1–2 min intervals with participants seated, legs uncrossed, after 5 min of rest; cuff size was matched to arm circumference, and the mean of three readings was used for analysis. BMI was calculated as weight in kilograms divided by the square of height in meters (kg/m^2^). A structured questionnaire was administered to collect information on lifestyle factors, including dietary habits such as sugar, salt, fat, and fruit and vegetable intake, physical activity, sleep quality, smoking, and alcohol consumption (Supplementary File S1). Responses were categorized as binary variables based on predefined thresholds: high sugar intake > 24 g/day [27], high sodium intake > 2 g/day [28], and high-fat dietary intake ≥ 3 times/week [29]. Inadequate fruit and vegetable intake < 5 servings/day, inadequate physical activity < 150 min/week, current smoking, and alcohol consumption within the past 12 months were assessed by self-report based on standard public health survey definitions [30]. Inadequate sleep duration was defined as <7 h/day based on National Sleep Foundation recommendations [31]. Personnel received standardized training, and all measurement instruments were calibrated according to manufacturer specifications.

### 2.3. Laboratory Analysis: PLASMA MDA Measurement

Venous blood samples were collected into EDTA anticoagulant tubes after a 12 h fast. Plasma was freshly prepared by centrifugation at 1500× *g* for 10 min using a centrifuge (N-TER, model ZN-1651, Guangzhou, China). Plasma MDA concentration was measured using the thiobarbituric acid reactive substances (TBARS) assay modified from Uchiyama, M. (1978) [32]. Briefly, 200 µL of plasma was combined with 750 µL 2.98% (*v*/*v*) orthophosphoric acid and 250 µL of 1.73% (*w*/*v*) thiobarbituric acid (TBA) solution. The mixture was incubated in a boiling water bath (95–100 °C) for 45 min to develop the MDA-TBA chromogen. Following the heating period, the reaction was stopped by placing the tubes in an ice bath. To remove any precipitated proteins or turbidity that might interfere with light scattering, the samples were centrifuged at 1500× *g* for 10 min. The absorbance of the clear supernatant was then measured using a UV-Vis spectrophotometer (DLAB, model V-1000, DLAB Scientific Co., Ltd., Beijing, China) at 532 nm. MDA concentrations were determined against a standard curve generated with 1,1,3,3-tetramethoxypropane and expressed as µmol/L of plasma. All samples were analyzed in triplicate, and the mean value was used for statistical analysis.

### 2.4. Reference Interval and Exploratory P75-Based Cutoff

Following the International Federation of Clinical Chemistry and Laboratory Medicine (IFCC) recommendations, the reference interval for plasma MDA was determined non-parametrically as the 2.5th to 97.5th percentiles (P2.5–P97.5) of the distribution in the healthy reference subgroup (*n* = 94). The corresponding 90% confidence intervals for each reference limit were estimated using the bootstrap percentile interval method (1000 resamples) [33]. In the absence of established outcome-based clinical cutoffs for plasma MDA in this population, participants with MDA levels at or above the 75th percentile (P75) of the reference distribution were defined as the exploratory P75-based elevated MDA subgroup. This percentile-based approach was chosen to increase sensitivity for detecting early-phase lipid peroxidation, consistent with exploratory methods widely adopted in epidemiological studies [34,35].

### 2.5. Statistical Analysis

All statistical analyses were performed using IBM SPSS Statistics for Windows, version 26.0 (IBM Corp., Armonk, NY, USA). Normality of continuous variables was assessed using the Kolmogorov–Smirnov test. Normally distributed data are presented as mean ± standard deviation (SD), and non-normally distributed data as median with interquartile range (IQR). Categorical variables were compared using the chi-square test (or Fisher’s exact test when expected cell counts were <5). Correlations between MDA levels and continuous variables were assessed using Spearman’s rank correlation coefficient for non-normally distributed data. Binary logistic regression was used to identify independent predictors of elevated MDA (≥P75). Variables with a *p*-value < 0.10 in univariate analysis (age, sex, BMI, high sugar intake, high-fat diet) were entered into the multivariate model using the Enter method. Adjusted odds ratios (OR) and 95% confidence intervals (CIs) were calculated. Model fit was assessed using the Hosmer–Lemeshow goodness-of-fit test, and the Nagelkerke *R*^2^ was reported. Plasma MDA concentration was also analyzed as a continuous outcome using multiple linear regression, following natural log-transformation to satisfy normality assumptions. The same covariates used in the logistic regression model were entered simultaneously. Unstandardized regression coefficients (B) with 95% confidence intervals were reported, and model fit was assessed using the adjusted *R*^2^. Collinearity was assessed using variance inflation factors (VIFs). A two-tailed *p*-value < 0.05 was considered statistically significant for all tests.

## 3. Results

### 3.1. Participant Characteristics and Plasma MDA Levels

A total of 141 college students (103 female, 73.0%; 38 male, 27.0%) with a mean age of 20.8 ± 1.7 years were enrolled. Baseline demographic, clinical, and lifestyle characteristics are summarized in Table 1. The median BMI was 21.2 kg/m^2^ (IQR 19.0–24.3), and the median waist circumference (WC) was 71 cm (IQR 66–80). Systolic and diastolic blood pressures were 121 ± 14.4 mmHg and 75 mmHg (IQR 70–82), respectively. Behavioral questionnaire data indicated that 73.0% of participants consumed more than 24 g of sugar per day, 51.8% reported a high-fat diet, 73.8% had inadequate fruit and vegetable intake, 84.4% engaged in insufficient physical activity, and 75.9% experienced poor sleep quality. The median plasma MDA concentration measured by the TBARS assay in the whole cohort was 4.32 µmol/L. In the healthy reference subgroup (*n* = 94), the distribution was non-normal (Kolmogorov–Smirnov test, *p* < 0.05). The 2.5th and 97.5th percentiles defined a reference interval of 1.16 (90% CI: 0.97–2.00) to 9.23 (90% CI: 7.97–9.92) µmol/L, and the 75th percentile (P75) was 6.07 µmol/L (Table 2). This P75 value was subsequently used as the cutoff to define the elevated MDA subgroup in all further analyses.

### 3.2. Univariate Associations with Elevated MDA

When the total cohort was divided into normal MDA (<6.07 µmol/L) and elevated MDA (≥6.07 µmol/L), 36 participants (25.5%) were classified into the elevated MDA subgroup. Chi-square tests revealed that high sugar intake and high-fat diet were significantly associated with elevated MDA (*p* = 0.013 and *p* = 0.038, respectively). Among participants who consumed more than 24 g of sugar daily, 31.1% had elevated MDA compared with 10.5% of those who did not, corresponding to an odds ratio (OR) of 3.83 (95% CI: 1.28–11.48). Similarly, 32.9% of individuals reporting a high-fat diet had elevated MDA versus 17.6% of those who did not (OR = 2.29; 95% CI: 1.04–5.02). Smoking showed a borderline association with elevated MDA (*p* = 0.072); however, this finding should be interpreted with caution given the small number of smokers in this cohort (*n* = 5). No other lifestyle factor (sex, physical inactivity, high sodium intake, fruit/vegetable consumption, alcohol consumption, or sleep quality) reached statistical significance, as shown in Table 3.

### 3.3. Correlation Analysis

Spearman’s rank correlation coefficients among continuous variables are presented in Table 4. Plasma MDA levels were significantly positively correlated with BMI (ρ = 0.19, *p* = 0.021). MDA showed no significant correlation with WC (ρ = 0.15, *p* = 0.076), systolic blood pressure (ρ = 0.05, *p* = 0.531), or diastolic blood pressure (ρ = 0.05, *p* = 0.502). As anticipated, BMI was strongly correlated with WC (ρ = 0.76, *p* < 0.001) and also significantly correlated with both systolic (ρ = 0.33, *p* < 0.001) and diastolic (ρ = 0.27, *p* = 0.001) blood pressures.

### 3.4. Multivariate Logistic Regression Analysis

To identify independent predictors of elevated plasma MDA, a binary logistic regression model was constructed including age, sex, BMI, high sugar intake, and high-fat diet (Table 5). After adjustment for all variables in the model, high sugar intake remained the only statistically significant independent predictor. Participants who consumed more than 24 g of sugar per day had a 3.42-fold higher odds of having elevated MDA compared with those who did not (adjusted OR = 3.42; 95% CI: 1.08–10.87; *p* = 0.036). Higher BMI showed a trend toward increased risk but did not reach statistical significance (adjusted OR = 1.07 per 1 kg/m^2^; 95% CI: 0.98–1.17; *p* = 0.128). Age had a borderline protective effect (adjusted OR = 0.76 per year; 95% CI: 0.55–1.04; *p* = 0.082). Sex (male vs. female) and high-fat diet were not independently associated with elevated MDA (*p* = 0.766 and *p* = 0.104, respectively). The Hosmer–Lemeshow test indicated good model fit (χ^2^(8) = 4.226, *p* = 0.623), and the Nagelkerke *R*^2^ was 0.142.

### 3.5. Multiple Linear Regression Analysis

To further examine the relationship between plasma MDA and its predictors, log-transformed plasma MDA concentration was analyzed as a continuous outcome using multiple linear regression (Table 6). After adjustment for the other covariates, only BMI was significantly associated with MDA (B = 0.028; 95% CI: 0.009–0.047; *p* = 0.004). Sugar intake was not significantly associated with MDA in this model (B = 0.139; 95% CI: −0.056 to 0.333; *p* = 0.162). Age, sex, and high-fat diet were also not significantly associated with MDA (*p* = 0.073, *p* = 0.900, and *p* = 0.371, respectively). All VIF values were below 1.1, indicating no meaningful multicollinearity. The adjusted *R*^2^ for the model was 0.064.

## 4. Discussion

To our knowledge, this is the first study to establish a preliminary, population-specific reference interval for plasma MDA measured by the TBARS-spectrophotometric method in Thai young adults, addressing a gap previously identified in the literature [18]. In this cross-sectional study of 141 Thai college students, a reference interval of 1.16 (90% CI: 0.97–2.00) to 9.23 (90% CI: 7.97–9.92) µmol/L was determined non-parametrically (P2.5–P97.5) following IFCC recommendations [18], using a healthy reference subgroup (*n* = 94)**.** Although Nielsen et al. [19] reported a plasma MDA reference interval of 0.36–1.24 µmol/L in a healthy Danish population using HPLC, direct application of these limits to our population and method may not be valid due to differences in ethnicity, age distribution, and dietary patterns [18]. Additionally, TBARS-based methods consistently yield higher MDA values than HPLC [20], further supporting the need for a method-specific and population-specific reference interval. The 75th percentile (P75 = 6.07 µmol/L) was subsequently used as an exploratory cutoff to classify participants with elevated MDA. This percentile-based approach is consistent with established epidemiological practice for risk stratification when outcome-based cutoffs are unavailable, as similarly applied in cardiovascular risk assessment [34,35]. Identifying individuals in the upper quartile of MDA distribution may support early detection of subclinical oxidative stress before the onset of clinically detectable metabolic disease, providing a rational basis for targeted preventive interventions at a stage when lifestyle modifications are most likely to be effective [16]. Using this exploratory cutoff, 25.5% of participants exceeded this threshold for elevated MDA levels, warranting further investigation into its relevance as a marker of oxidative stress among apparently healthy Thai young adults. This finding is consistent with growing evidence that lifestyle-induced oxidative stress is prevalent even before overt metabolic disease becomes detectable [9,36]. After adjusting for age, sex, BMI, and high-fat diet, high sugar intake remained independently associated with elevated MDA (adjusted OR = 3.42; 95% CI: 1.08–10.87; *p* = 0.036). Although univariate analyses showed significant associations for both high sugar intake and high-fat diet, the latter lost significance in the multivariate logistic model. These findings highlight that daily sugar intake exceeding 24 g (approximately 6 teaspoons) is independently associated with elevated lipid peroxidation in young, generally lean adults, even after adjustment for adiposity and other lifestyle factors.

Our findings are consistent with previous reports linking dietary sugar intake to elevated oxidative stress markers. Cross-sectional evidence has demonstrated that higher dietary sugar intake is independently associated with elevated plasma MDA concentrations in adults at risk of metabolic syndrome [37]. This finding supports the notion that dietary sugar is a modifiable contributor to lipid peroxidation, and several plausible mechanisms may underlie this association. In Thai young adults, dietary sugar is largely derived from sucrose-containing beverages and foods, providing both fructose and glucose as oxidative substrates [38]. Fructose is rapidly metabolized in the liver, independent of insulin, generating uric acid and depleting ATP, which directly stimulates mitochondrial ROS production and subsequent lipid peroxidation, a pathway documented in humans consuming fructose-rich diets regardless of metabolic disease status [39]. Additionally, high-sugar meals induce postprandial oxidative stress through mitochondrial superoxide overproduction even in non-diabetic individuals [40]. More broadly, excess energy intake in the form of high-fat, high-carbohydrate meals has been shown to increase MDA levels, even in healthy, lean individuals, despite compensatory antioxidant enzyme upregulation [41]. These mechanisms converge on PUFA peroxidation and MDA formation, consistent with our TBARS-based findings, though direct mechanistic evidence was not assessed in this study. Our observation that sugar intake at levels approaching current public health recommendations was associated with elevated MDA in the logistic model suggests that even moderate dietary sugar consumption may be associated with measurable oxidative damage in young, apparently healthy individuals. In contrast, the univariate association between high-fat diet and elevated MDA did not survive multivariate adjustment. This is consistent with evidence that the pro-oxidant effect of high-fat diets may be primarily mediated through obesity-induced adipose tissue dysfunction and insulin resistance, rather than representing a direct effect of dietary fat per se [42]. In our study, BMI and waist circumference were strongly correlated (ρ = 0.765, *p* < 0.001), and the inclusion of BMI in the regression model likely captured part of the effect of high-fat diet. Moreover, the TBARS assay preferentially detects lipid peroxidation from polyunsaturated fatty acids (PUFAs) and is less sensitive to saturated fats, which may also explain the lack of an independent association [19]. Despite a significant positive correlation between BMI and plasma MDA (ρ = 0.194, *p* = 0.021), BMI was not independently associated with elevated MDA in the multivariate logistic model, which may be partly attributable to the narrow BMI distribution of our sample (median 21.2 kg/m^2^; IQR 19.0–24.3), consistent with evidence that BMI-MDA associations are most apparent in studies incorporating wider BMI ranges that include overweight or obese individuals [43]. The attenuation of BMI in the multivariate logistic model may also partly reflect its correlation with sugar intake; notably, sugar intake retained independent significance after BMI adjustment (adjusted OR = 3.42; *p* = 0.036), consistent with previous evidence that dietary sugar is independently associated with plasma MDA beyond adiposity [37]. Plasma MDA was additionally analyzed as a continuous, log-transformed outcome using multiple linear regression (Table 6). BMI emerged as the only variable significantly linked to MDA (B = 0.028; 95% CI: 0.009–0.047; *p* = 0.004), whereas sugar intake was not (B = 0.139; *p* = 0.162). The recovery of a significant BMI–MDA association is consistent with the well-documented statistical cost of dichotomizing continuous variables, which reduces power and may obscure genuine linear associations [44,45]. A comparable loss of a statistically significant association upon dichotomizing a continuous biomarker at a fixed cutoff has been demonstrated for other biomarkers, such as the neutrophil-to-lymphocyte ratio in oncology research [46], and is further supported by the univariate correlation between BMI and MDA (ρ = 0.194, *p* = 0.021), as well as prior reports in other populations [43]. Conversely, the loss of significance for sugar intake may reflect the circularity inherent to the P75 cutoff, a well-documented phenomenon in studies using data-derived cutpoints [47,48]; this non-significant result does not itself rule out a true but small effect [49]. Nonetheless, the modest effect size and explanatory power of this model (adjusted *R*^2^ = 0.064) indicate that BMI alone accounts for only a small proportion of the variance in plasma MDA, consistent with the multifactorial nature of oxidative stress biomarkers. Together, these two analytic approaches offer complementary insights: the P75-based model identifies sugar intake as an exploratory indicator for categorical risk stratification, while the continuous-outcome model highlights BMI as a statistically robust correlate of lipid peroxidation across the full range of adiposity. The P75-based cutoff provides a practical tool for prioritizing young adults for further evaluation of oxidative stress-related lifestyle factors. No universal clinical decision limit for plasma MDA exists; however, percentile-based risk stratification is widely accepted in epidemiological studies of oxidative stress biomarkers. Cesena et al. demonstrated that Brazilian adults at or above the 75th percentile of 10-year ASCVD risk had 6.6- to 11.6-fold higher estimated long-term cardiovascular risk [35], while Wang et al. confirmed that demographic-specific P75 of coronary artery calcium is a recommended high-risk threshold in clinical guidelines [34]. We acknowledge this as an exploratory approach and recommend future prospective studies to validate the clinical utility of this cutoff in larger and more diverse populations. Although the TBARS assay has analytical limitations, including low specificity for MDA due to cross-reactivity with other aldehydes and lipid peroxidation products [50], its simplicity and cost-effectiveness make it feasible for large-scale epidemiological screening. Notably, plasma MDA concentrations rise significantly following glucose ingestion in postprandial states [51]. Blood samples in this study were therefore collected after a 12 h fast to minimize this potential source of interference. Nonetheless, cross-reactivity with reducing sugars such as sucrose, fructose, and glucose cannot be entirely excluded. As the sugar intake variable reflected habitual dietary pattern rather than the sugar concentration of the assayed sample itself, the observed association more plausibly reflects a chronic metabolic pathway of sugar-induced oxidative stress than an acute in-assay artifact. This interpretation, however, remains hypothetical and does not by itself resolve the assay’s inherent specificity limitation. In contrast to hsCRP, which reflects downstream inflammation and may lack sensitivity in apparently healthy populations [16], MDA directly measures upstream oxidative damage and showed significant associations with modifiable lifestyle factors in this study, supporting its potential utility as an exploratory screening biomarker in young adults. The World Health Organization recommends limiting added sugar intake to less than 5% of total energy intake, equivalent to approximately 25 g per day, consistent with the threshold adopted in this study [27].

Strengths of this study include (i) the establishment of a population-specific plasma MDA reference interval in Thai young adults following IFCC recommendations, providing a practical foundation for population-based oxidative stress surveillance and NCD prevention strategies targeting young adults at a stage when lifestyle interventions are most effective [16,36]; (ii) use of multivariate logistic regression controlling for multiple lifestyle confounders, with good model fit confirmed by the Hosmer–Lemeshow test (*p* = 0.623); and (iii) use of the TBARS-based MDA assay, which offers high sensitivity, reproducibility, and technical simplicity, making it well suited to population-level oxidative stress screening, with potential applicability to individual monitoring over time [17], particularly in resource-limited university health settings. Despite these strengths, some limitations of the present study should be considered. First, the cross-sectional design precludes causal inference, and unmeasured confounders, including antioxidant intake, undiagnosed inflammatory conditions, and genetic predisposition, cannot be fully excluded. Second, the modest sample size and limited number of participants with elevated MDA may have restricted statistical power, potentially contributing to the non-significant findings for BMI and high-fat diet in the multivariate logistic model. In addition, subgroup analysis by health status further reduced group sizes, limiting the power to detect weaker associations. Third, lifestyle exposures were assessed by self-report, which may be subject to recall bias. Fourth, the predominance of female participants (73.0%) may limit generalizability to male university students. Given the limited number of male participants (*n* = 38), a sex-stratified analysis was not performed, and the observed non-significant association between sex and elevated MDA (*p* = 0.317) may partly reflect limited statistical power. Estrogen’s antioxidant effects have been proposed to lower baseline MDA in premenopausal women, based on the decline in estrogen levels after menopause [52], and may be particularly relevant to this young, premenopausal cohort. As the reference interval in this study was derived predominantly from female participants, caution is warranted in extending these values to represent male populations. Additionally, as participants were recruited from a single-center population, the reference interval may not be generalizable to other institutions or geographic regions within Thailand. Fifth, the P75 cutoff, derived from the reference interval, was subsequently evaluated for its associations with lifestyle and cardiometabolic factors in the same cohort from which the reference subgroup itself originated. This introduced potential selection bias, as evaluating cutoff performance in the same population used for its derivation may yield an overly optimistic estimate of discriminatory accuracy [46], a concern addressed through the complementary multiple linear regression analysis presented above (Table 6). As this study represents an initial step toward establishing a reference interval for this biomarker, the resulting cutoff may be subject to a spectrum effect, whereby these associations vary across subgroups differing in underlying health status [53]. Accordingly, this cutoff should be regarded as an exploratory threshold that requires prospective validation, stratified by health status and in larger, more age-diverse populations, before broader clinical or screening application. Sixth, because the reference sample size (*n* = 94) fell below the minimum of 120 individuals recommended by CLSI for stable non-parametric confidence interval estimation [33], the resulting confidence intervals around the reference limits were relatively wide. This finding further supports the characterization of the reported values as a preliminary reference interval. The findings of this study have potential implications for early NCD prevention strategies targeting young adults. If validated prospectively, plasma MDA measured by the TBARS assay could serve as a practical, low-cost screening tool for identifying apparently healthy young adults with elevated oxidative stress markers, enabling timely lifestyle interventions before irreversible cardiometabolic sequelae develop. Several directions for future research are warranted. First, prospective cohort studies are needed to examine whether elevated plasma MDA independently predicts future metabolic disease development in young adults, thereby establishing the prognostic value of this biomarker beyond cross-sectional associations. Second, longitudinal studies tracking plasma MDA levels in apparently healthy young adults over time would help determine whether persistently elevated MDA predicts the subsequent development of metabolic abnormalities, thereby establishing its predictive validity as an early screening biomarker at the individual level. Third, future studies should simultaneously measure MDA alongside low-grade inflammatory biomarkers to confirm this proposed relationship, which is a prerequisite for evaluating MDA-based classification as a screening biomarker, with preference for accessible and cost-effective markers suitable for large-scale screening. Fourth, replication using more analytically specific methods such as HPLC or GC-MS would help confirm the associations observed with the TBARS assay. Fifth, future studies should measure dietary intake continuously rather than by categorical thresholds, enabling formal dose–response analysis and substantiating the association between sugar intake and elevated oxidative stress markers. Finally, the preliminary reference interval established in this study should be validated in larger, geographically diverse Thai populations with a more balanced sex distribution. Ultimately, incorporating oxidative stress surveillance into routine university health programs may represent a cost-effective and scalable approach to NCD prevention in young Thai adults.

## 5. Conclusions

In summary, this study established a preliminary, population-specific reference interval for plasma MDA of 1.16–9.23 µmol/L in healthy Thai young adults using the TBARS-spectrophotometric method following IFCC recommendations for oxidative stress assessment in this population. Using the 75th percentile (P75 = 6.07 µmol/L) as an exploratory cutoff, 25.5% of participants exceeded this study-derived threshold for elevated MDA levels, warranting further investigation into its relevance as a marker of oxidative stress in this apparently healthy Thai undergraduate population. High sugar intake exceeding 24 g/day was independently associated with elevated plasma MDA in the P75-based logistic model, highlighting dietary sugar as a modifiable factor linked to lipid peroxidation and, potentially, early low-grade chronic inflammation in young adults. However, when plasma MDA was analyzed as a continuous outcome, BMI, rather than sugar intake, was independently associated with MDA. These results likely reflect complementary exploratory associations for these two factors, captured through categorical versus continuous analysis, rather than independent confirmation of causal relationships, warranting further investigation in larger, independent samples. The TBARS-based MDA assay may serve as a practical and cost-effective tool for population-level oxidative stress screening in resource-limited settings. These findings highlight the potential relevance of limiting added sugar consumption from early adulthood as a strategy that may help address oxidative stress burden and long-term cardiometabolic disease risk. Further prospective longitudinal studies are warranted to confirm these findings and to evaluate the clinical utility of plasma MDA, including the discriminatory value of the P75 cutoff, as a candidate screening biomarker for early oxidative stress surveillance in young, apparently healthy populations.

## Figures and Tables

**Table 1:** Demographic, clinical, and lifestyle characteristics of enrolled students.

Parameters	Value
Demographic and clinical characteristics	
Total enrolled students (*n*)	141
Age (years)	20.8 ± 1.7
Gender, *n* (%)	
Male	38 (27.0)
Female	103 (73.0)
Body mass index (kg/m^2^)	21.2 (19.0–24.3)
Systolic blood pressure (mmHg)	121 ± 14.4
Diastolic blood pressure (mmHg)	75 (70–82)
Waist circumference (cm)	71 (66–80)
MDA (µmol/L)	4.32 (3.12–6.14)
Lifestyle factors, *n* (%)	
High-sugar dietary intake	103 (73.0)
High-sodium dietary intake	64 (45.4)
High-fat dietary intake	73 (51.8)
Inadequate fruit and vegetable intake	104 (73.8)
Inadequate physical activity	119 (84.4)
Poor sleep quality	107 (75.9)
Smoking	5 (3.5)
Alcohol consumption	91 (64.5)

Values are presented as mean ± SD for normally distributed and as median (IQR: 25–75th percentile) for non-normally distributed variables. Normality was assessed using the Kolmogorov–Smirnov test (*p* < 0.05). Abbreviations: IQR, interquartile range; SD, standard deviation.

**Table 2:** Reference intervals for plasma MDA (*N* = 94).

Biomarker	Mean ± SD	Median (IQR)	P2.5 (90% CI)	P97.5 (90% CI)	Reference Intervals
MDA (µmol/L)	4.56 ± 2.00	4.23 (3.14–6.07)	1.16 (0.97–2.00)	9.23 (7.97–9.92)	1.16–9.23

The distribution of the data was found to be non-normal (*p* < 0.05, Kolmogorov–Smirnov test). Reference intervals were calculated according to the IFCC guidelines using a non-parametric method. Mean ± SD is presented for descriptive comparison only and was not used to derive the reference interval.

**Table 3:** Association between lifestyle factors and high plasma MDA levels (≥75th percentile, cutoff = 6.07 µmol/L).

Parameter	Total (*N* = 141)	MDA < 6.07*n* (%)	MDA ≥ 6.07*n* (%)	*p*-Value	OR (95% CI)
Sex				0.317	0.66 (0.29–1.48)
Male	38	26 (68.4)	12 (31.6)		
Female	103	79 (76.7)	24 (23.3)		
High sugar dietary intake				0.013	3.83 (1.28–11.48)
No	38	34 (89.5)	4 (10.5)		
Yes	103	71 (68.9)	32 (31.1)		
High sodium dietary intake				0.302	1.49 (0.70–3.16)
No	77	60 (77.9)	17 (22.1)		
Yes	64	45 (70.3)	19 (29.7)		
High fat dietary intake				0.038	2.29 (1.04–5.02)
No	68	56 (82.4)	12 (17.6)		
Yes	73	49 (67.1)	24 (32.9)		
Inadequate fruit and vegetable intake				0.525	1.336 (0.546–3.26)
No	37	29 (78.4)	8 (21.6)		
Yes	104	76 (73.1)	28 (26.9)		
Inadequate physical activity				0.838	0.90 (0.33–2.48)
No	22	16 (72.7)	6 (27.3)		
Yes	119	89 (74.8)	30 (25.2)		
Current smoking				0.072	4.68 (0.74–29.7)
No	136	103 (75.7)	33 (24.3)		
Yes	5	2 (40.0)	3 (60.0)		
Alcohol consumption				0.925	0.97 (0.44–2.12)
No	50	37 (74.0)	13 (26.0)		
Yes	91	68 (74.7)	23 (25.3)		
Poor sleep quality				0.295	1.61 (0.69–3.78)
No	34	23 (67.6)	11 (32.4)		
Yes	107	82 (76.6)	25 (23.4)		

Data are presented as *n* (%). *p*-values were calculated using the Chi-square test for categorical variables. The cutoff for “high” MDA levels was defined as the 75th percentile of the distribution (≥6.07 µmol/L). OR, odds ratio; CI, confidence interval; MDA, malondialdehyde. Odds ratios and 95% CI calculated for the exposure “Yes” vs. “No” (reference = “No”).

**Table 4:** Correlation between plasma MDA levels and cardiometabolic parameters.

Variable	MDA (µmol/L)	BMI (kg/m^2^)	WC (cm)	SBP (mmHg)	DBP (mmHg)
MDA (µmol/L)	1.00	0.19 (0.021) *	0.15 (0.076)	0.05 (0.531)	0.06 (0.502)
BMI (kg/m^2^)		1.00	0.77 (<0.001) **	0.33 (<0.001) **	0.28 (0.001) **
WC (cm)			1.00	0.35 (<0.001) **	0.24 (0.004) **
SBP (mmHg)				1.00	0.43 (<0.001) **
DBP (mmHg)					1.00

* *p* < 0.05; ** *p* < 0.01. Values are Spearman’s rho (*p*-value). Abbreviations: MDA, malondialdehyde; BMI, body mass index; WC, waist circumference; SBP, systolic blood pressure; DBP, diastolic blood pressure. Note: Lower triangle of the symmetric matrix is shown; upper triangle (empty cells) is omitted for clarity.

**Table 5:** Multiple logistic regression analysis of factors associated with elevated plasma MDA levels (≥75th percentile, cutoff = 6.07 µmol/L) among college students (*N* = 141).

Variable	Adjusted OR	95% CI	*p*-Value
Age (per year)	0.76	0.55–1.04	0.082
Sex (male vs. female)	1.15	0.46–2.87	0.766
BMI (per kg/m^2^)	1.07	0.98–1.17	0.128
High sugar intake (yes vs. no)	3.42	1.08–10.87	0.036
High-fat diet (yes vs. no)	2.00	0.87–4.61	0.104

Abbreviations: OR, odds ratio; CI, confidence interval; MDA, malondialdehyde; BMI, body mass index. Model summary: Nagelkerke *R*^2^ = 0.142; Hosmer–Lemeshow goodness of fit test *p* = 0.623. Variables were entered using the Enter method. Reference categories: female, no high sugar intake, no high-fat diet. The dependent variable was elevated MDA (≥6.07 µmol/L, 75th percentile).

**Table 6:** Multiple linear regression analysis of factors associated with log-transformed plasma MDA concentration among college students (*N* = 141).

Variable	B	95% CI	*p*-Value
Age (per year)	−0.047	−0.099 to 0.004	0.073
Sex (male vs. female)	−0.013	−0.212 to 0.186	0.900
BMI (per kg/m^2^)	0.028	0.009–0.047	0.004
High sugar intake (>24 g/day) (yes vs. no)	0.139	−0.056 to 0.333	0.162
High-fat diet (yes vs. no)	0.079	−0.095 to 0.254	0.371

Abbreviations: B, unstandardized regression coefficient; CI, confidence interval; MDA, malondialdehyde; BMI, body mass index. Model summary: adjusted *R*^2^ = 0.064. Variables were entered simultaneously. Reference categories: female, no high sugar intake, no high-fat diet. The dependent variable was natural log-transformed plasma MDA concentration.

## Data Availability

The data presented in this study are available on request from the corresponding author due to privacy restrictions.

## Supplementary Materials

The following supporting information can be downloaded at: https://www.mdpi.com/article/10.3390/diagnostics16172697/s1, File S1. Research Questionnaire.

## Abbreviations

The following abbreviations are used in this manuscript:

MDA	Malondialdehyde
NCDs	Non-communicable diseases
ASCVD	Atherosclerotic cardiovascular disease
TBARS	Thiobarbituric acid reactive substances
IFCC	International Federation of Clinical Chemistry and Laboratory Medicine
hsCRP	High-sensitivity C-reactive protein
ROS	Reactive oxygen species
BMI	Body mass index
WC	Waist circumference
SBP	Systolic blood pressure
DBP	Diastolic blood pressure
OR	Odds ratio
CI	Confidence interval
IQR	Interquartile range
SD	Standard deviation
