# Supplementary material for: Plasma Malondialdehyde Reference Interval and Its Associations with Lifestyle Risk Factors in Thai Undergraduate Students: A Single-Center Cross-Sectional Study
Source: Diagnostics (Basel). 2026 Aug 24;16(17):2697. doi: 10.3390/diagnostics16172697 (PMC13565091; doi:10.3390/diagnostics16172697)
Supplement: Supplementary file 1 [file diagnostics-16-02697-s001.zip › diagnostics-4405206-supplementary.pdf]

Research Questionnaire

Supplementary File S1

General Instructions

Dear Respondent,

1. This questionnaire was developed solely for research purposes.
2. Please answer every item truthfully, in accordance with your actual opinions, feelings, and behaviors, to ensure accurate and valid research findings and to support future development.
3. Every response you provide is valuable and directly contributes to this research. All information obtained from this questionnaire will be kept confidential and will not affect you or your organization in any way.

General Information

Instructions: Please obtain the following information from the study participant and record it in the space provided, or mark (✓) the appropriate box as applicable.

Name–Surname: ____

Sex* ☐ Male ☐ Female

Age (years)*: ____________ Weight: ____________ kg Height: ____________ cm

BMI: ____________ kg/m²

Mean blood pressure (SYS/DIA): ____________ mmHg

Waist circumference: ____________ cm

Do you have a history of any chronic disease?

☐ No chronic disease
☐ Hypertension
☐ History of diabetes mellitus
☐ History of cardiovascular or cerebrovascular disease
☐ Other, please specify: ______________________

Instructions: Please mark (✓) the box that best matches your opinion for each item.

Part 1: Physical Activity

1.1 How often do you exercise per week?

☐ Never ☐ 1–2 times/week
☐ 3 times/week ☐ 4–5 times/week or more

1.2 On each occasion, approximately how many minutes do you exercise?

☐ Less than 15 minutes ☐ 15–30 minutes
☐ 30–60 minutes ☐ More than 60 minutes

Note: *A total of less than 150 minutes/week is considered inadequate physical activity.*

Part 2: Dietary Intake

2.1 How often do you consume sweets, sweetened drinks, soft drinks, or beverages containing 6 teaspoons of sugar or more?

☐ Never ☐ 1–2 times/week
☐ 3–4 times/week ☐ Every day

Note: *Consumption at a frequency of 3 times/week or more is considered indicative of sugar intake exceeding 24 g/day (6 teaspoons).*

2.2 How often do you consume salty foods such as spicy salads (yum), papaya salad (som tam), pickled foods, Thai-style barbecue (moo kra-ta), shabu-shabu, or instant noodles?

☐ Never ☐ 1–2 times/week

☐ 3–4 times/week ☐ Every day

Note: *Consumption at a frequency of 3 times/week or more is considered indicative of sodium intake exceeding 2 g/day.*

2.3 How often do you consume high-fat foods such as fried food, three-layer pork belly, or coconut-milk curry?

☐ Never ☐ 1–2 times/week

☐ 3–4 times/week ☐ Every day

Note: *Consumption at a frequency of 3 times/week or more is considered high-fat intake.*

2.4 How often do you eat at least 5 servings/day of fruits and vegetables (1 serving ≈ 1 scoop [tapoo] of vegetables, or one fist-sized piece of fruit, e.g., 1 apple)?

☐ Do not eat fruits/vegetables ☐ Occasionally (fewer than 5 servings/day)

☐ Every day (at least 5 servings/day)

Note: *Consumption of fewer than 5 servings/day of fruits and vegetables is considered inadequate.*

Part 3: Smoking

3.1 In the past 12 months, have you smoked?

☐ Never smoked ☐ Former smoker (quit)

☐ Smoke occasionally ☐ Smoke every day

3.2 If you smoke every day, approximately how many cigarettes do you smoke per day?

☐ 1–5 cigarettes ☐ 6–10 cigarettes

☐ 11–20 cigarettes ☐ More than 20 cigarettes

Note: *Smoking even occasionally within the past 12 months is classified as current smoking.*

Part 4: Alcohol Consumption

4.1 In the past 12 months, have you consumed alcoholic beverages?

☐ Never ☐ Former drinker (quit)

☐ Occasionally ☐ Weekly, on a regular basis

4.2 If you drink weekly on a regular basis, approximately how many times per week do you drink?

☐ 1–2 times/week ☐ 3–4 times/week

☐ 5 times/week or more

Note: *Consuming alcohol even occasionally within the past 12 months is classified as alcohol consumption.*

Part 5: Sleep/Rest

5.1 How many hours do you sleep per day?

☐ Less than 4 hours/day ☐ 5–6 hours/day

☐ 7–8 hours/day ☐ More than 8 hours/day

Note: *Sleeping 7 hours or more per day is considered adequate rest.*

Reference: The classification criteria in this questionnaire are based on standards from the World Health Organization (WHO) and the National Sleep Foundation (NSF).
